# Cardiomegaly With an Absent Tricuspid Septal Leaflet: A Cadaveric Case Report

**DOI:** 10.7759/cureus.106384

**Published:** 2026-04-03

**Authors:** Darrow Felsted, Alex King, Kyle Bergfalk, Schafer Paladichuk, Christian Heck

**Affiliations:** 1 Department of Anatomy, Pacific Northwest University of Health Sciences, Yakima, USA; 2 Department of Anatomy, Pacific Northwest University of Health Sciences - College of Osteopathic Medicine, Yakima, USA

**Keywords:** absent leaflet, cadaver case report, cardiomegaly, cardiovascular, tricuspid valve insufficiency

## Abstract

The heart functions as a coordinated four-chambered pump, with four valves ensuring unidirectional blood flow. The tricuspid valve, located between the right atrium and right ventricle, consists of anterior, posterior, and septal leaflets that coapt during systole to prevent retrograde flow. Valvular disorders may result in stenosis or regurgitation, thereby increasing cardiac workload and predisposing to chamber dilation and heart failure. Congenital abnormalities, including partial or complete leaflet absence, are rare but can significantly disrupt normal hemodynamics. Reports of absent tricuspid valve leaflets are exceedingly uncommon and have primarily involved the anterior or posterior leaflet.

During the thoracic dissection of a 41-year-old female body donor with a reported history of cardiovascular disease, cardiomegaly was identified. Gross examination revealed marked right atrial and ventricular enlargement. Internal inspection demonstrated a complete absence of the septal leaflet of the tricuspid valve, with intact anterior and posterior leaflets. Additional findings included dilation of the superior and inferior venae cavae, hypertrophied right atrial pectinate muscles, smooth and flattened right ventricular trabeculae carneae, and heterogeneous right ventricular myocardial thickness. The left heart structures were largely unremarkable aside from calcification of the mitral valve.

Isolated absence of the septal tricuspid leaflet in adulthood has not been previously described. Loss of septal leaflet coaptation likely resulted in chronic tricuspid regurgitation, progressive right-sided volume overload, compensatory chamber dilation, and eventual cardiomegaly. Recognition of such rare structural variants is important for accurate imaging interpretation and surgical planning, particularly given potential distortion of critical landmarks such as the Triangle of Koch.

## Introduction

Though seemingly simple in structure, the heart functions as a dynamic four-chambered pump that sustains both systemic and pulmonary circulation through coordinated mechanical activity. Four valves maintain unidirectional blood flow through the heart, ensuring efficient forward propulsion of blood. Atrioventricular valves are positioned between atria and ventricles and prevent retrograde flow during ventricular systole. These valves consist of fibrous leaflets extending into the lumen of the atrioventricular orifice from attachments to the fibrous skeleton of the heart. The free edges of each leaflet are attached to tendinous cords, which in turn attach to papillary muscles extending off the ventricular wall. The right atrioventricular valve, also known as the tricuspid valve, is the first valve encountered by venous return (deoxygenated blood) as it flows from the right atrium into the right ventricle. The valve is composed of three leaflets (anterior, posterior, and septal), which coapt during systole to facilitate forward blood flow into the pulmonary circulation [1]. Dysfunction of a leaflet or component of the tricuspid valve may lead to incomplete closure and, thus, regurgitation of blood into the right atrium.

Valvular disorders encompass a broad spectrum of structural and functional abnormalities that impair normal hemodynamics. These conditions may result in stenosis, regurgitation, or a combination of both, ultimately increasing cardiac workload and predisposing patients to chamber dilation and heart failure [1-2]. Etiologies include congenital malformations, degenerative processes, infectious causes, and pulmonary hypertension. When left unrecognized or untreated, valvular dysfunction can lead to progressive cardiac remodeling and irreversible myocardial damage [3-5]. In rare instances, structural abnormalities may extend to partial or complete absence of a valvular leaflet [4-7].

Absent valvular leaflets disrupt normal leaflet coaptation and permit significant regurgitant flow [3-4,7-10]. Chronic retrograde blood flow can increase chamber volume load, leading to progressive dilation and compensatory remodeling [8,11]. Absent leaflets are extremely rare and are associated with conditions such as Ebstein’s anomaly, tricuspid dysplasia, or leaflet agenesis in addition to iatrogenic causes [10,12-13]. A very limited number of cases describing congenital tricuspid valve leaflet agenesis have been reported, most commonly involving either the anterior or posterior leaflets [3-4,6-8,10]. Such structural absence may ultimately contribute to severe tricuspid regurgitation and subsequent chamber enlargement, including the development of cardiomegaly. In this report, we describe an absent septal leaflet in a 41-year-old female cadaveric donor with cardiomegaly.

## Case presentation

An enlarged heart was initially discovered in a 41-year-old female cadaveric donor during routine thoracic dissection for a university clinical anatomy course. The rights of the donor were protected, and past medical history was limited to the cause of death: cardiovascular disease. The cadaver had five observable sternal wires; however, the specific cardiac procedure could not be identified. No other surgical procedures or medical implants were observed on the thoracic wall or heart exterior.

**Figure 1:**
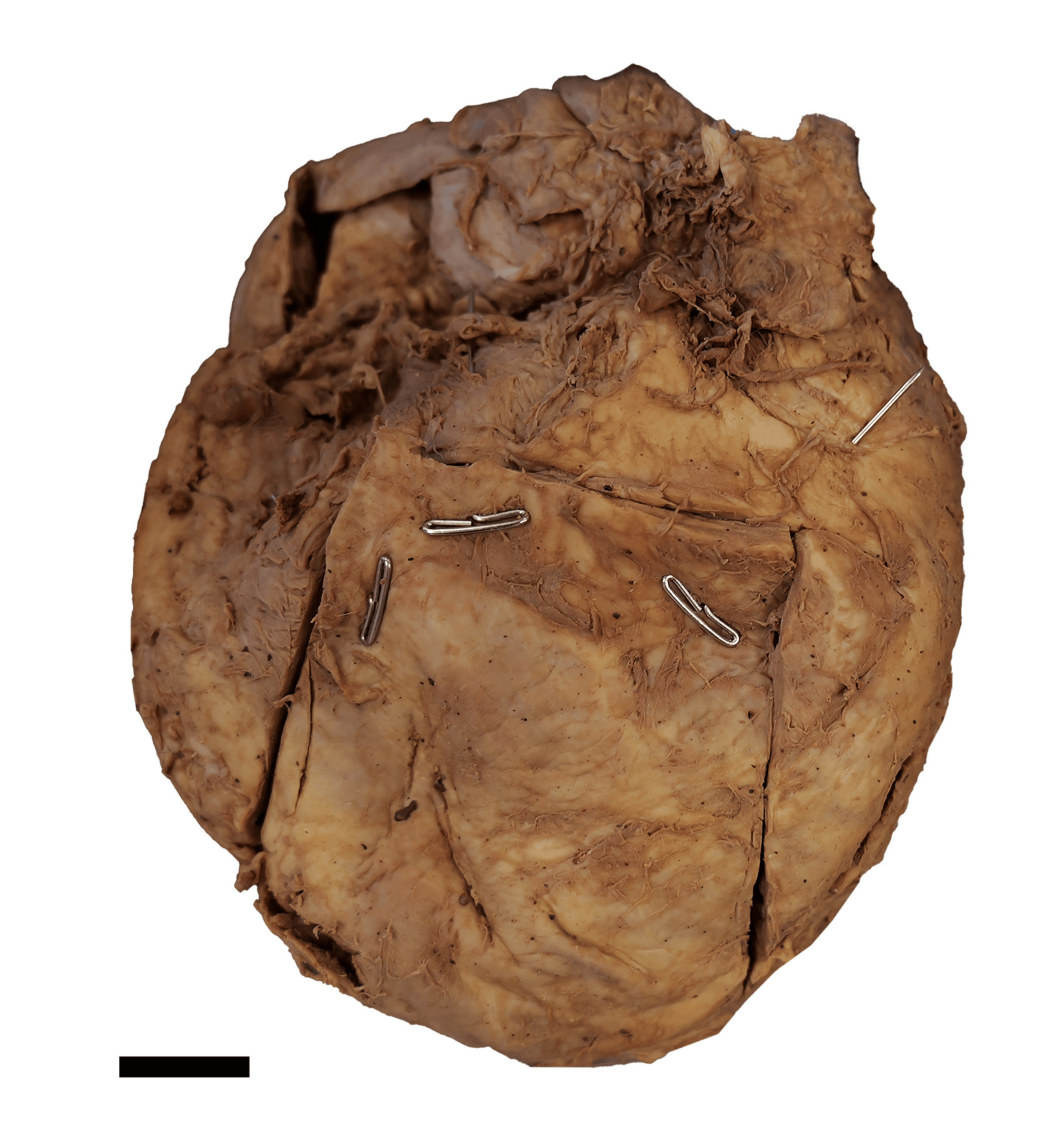
Anterior gross image of heart explanted from the 41-year-old body donor The incised window (outlined) demonstrates the right ventricle. Scale bar = 2 cm

**Figure 2:**
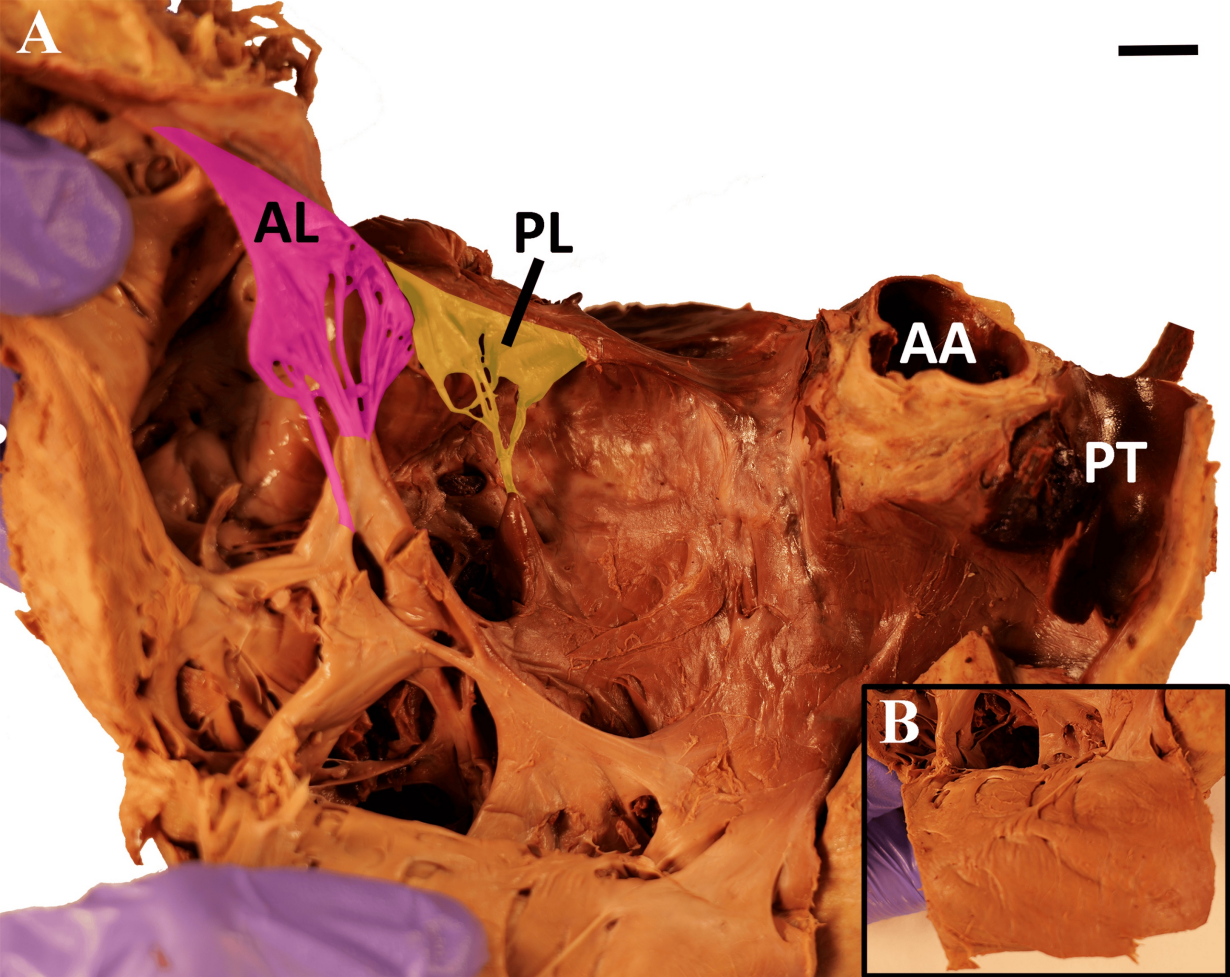
(A) Incised right ventricle including tricuspid valve with anterior leaflet (AL) and posterior leaflet (PL), but no visible septal leaflet. (B) Internal anterior right ventricular wall displaying smooth and flattened trabeculae carneae Scale bar = 1 cm AA: ascending aorta; PT: pulmonary trunk

To secure an appropriate view of the heart, the anterior chest wall was removed, and the lungs were resected at the pulmonary roots. The heart was excised from the chest by severing the great vessels approximately 2 cm distal to the heart. Initial measurements demonstrated a maximum transverse diameter of 14 cm from the apex to the lateral-most edge of the right atrial appendage, following the protocol outlined by Shimizu and Minamino [11]. Windows were cut into each heart chamber (Figure 1), and each atrioventricular valve was opened via scalpel incision (Figure 2A).

**Figure 3:**
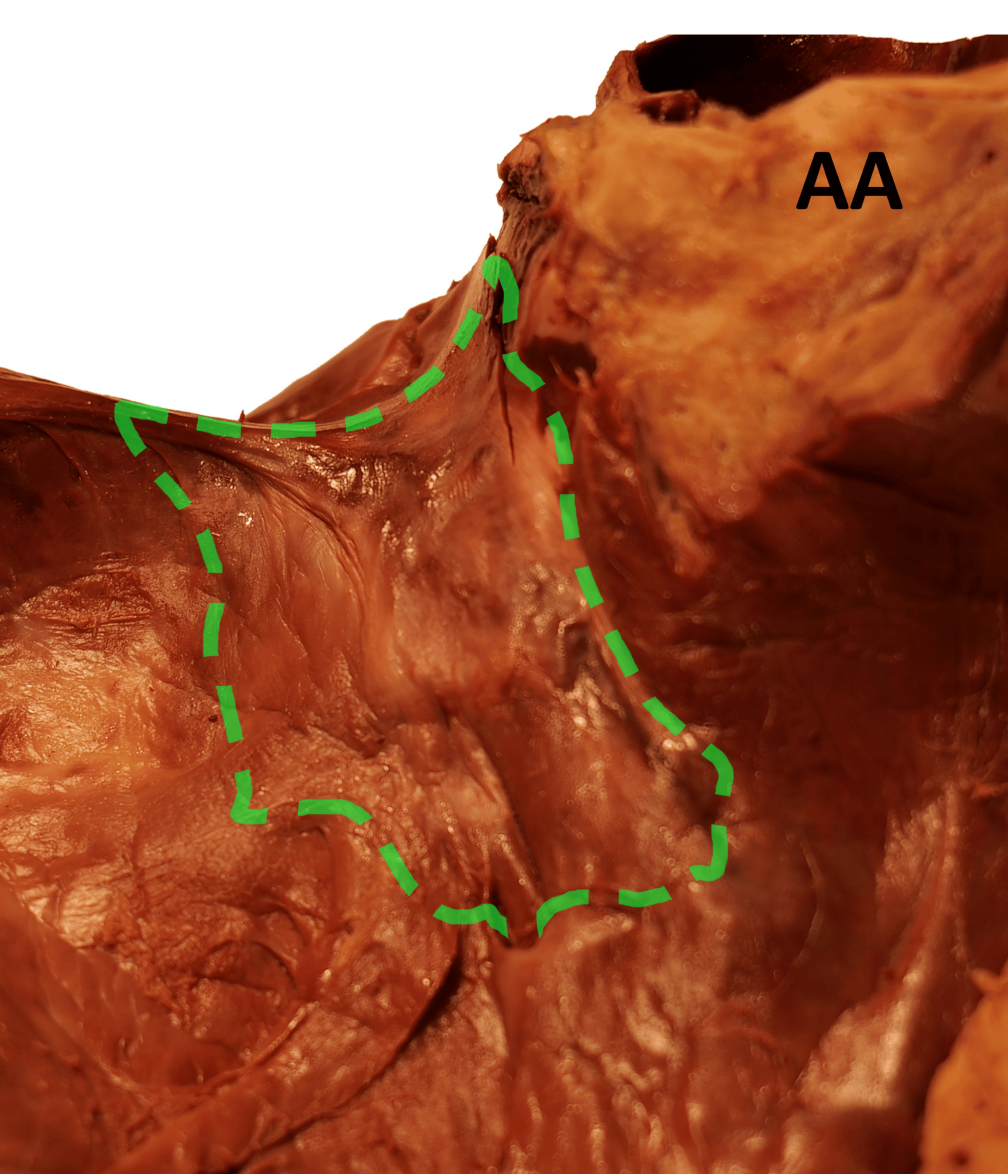
Focused examination of absent septal leaflet of tricuspid valve (green outline) AA: ascending aorta

Incisions revealed an unusually large right atrium (RA) and right ventricle (RV) in addition to an absent septal leaflet of the tricuspid valve. The tricuspid valve was not displaced, and atrialization of the right ventricle was not observed. The remaining anterior and posterior leaflets were without enlargement or fusion, resulting in an open space where the septal leaflet typically resides (Figure 3). Septal papillary muscles and associated chordae tendineae were also absent from the RV (Figures 2A, 3). Surgical artifacts and lesions were not observed in the internal heart.

Additional anatomical observations of the heart included smooth and flattened RV trabeculae carneae and diffuse thinning of the RV myocardium (Figures 2A-2B). The left side of the heart was without signs of septal defects or hypertrophy. However, there was evidence of calcium deposition within the anterior and posterior leaflets of the mitral valve. Finally, the lung parenchyma appeared grossly normal.

## Discussion

Cardiomegaly, defined as a maximum transverse cardiac diameter exceeding 13 cm in females and 15 cm in males, is associated with numerous etiologies, including valvular disorders and congenital abnormalities, each of which may increase the risk of heart failure [11,14-15]. The female body donor's heart, in this case, demonstrated a maximum transverse diameter of 14 cm, meeting criteria for cardiomegaly. Gross examination revealed a complete absence of the septal leaflet of the tricuspid valve and associated subvalvular apparatus (Figure 3). Absent tricuspid leaflets are associated with numerous disorders, including Ebstein’s anomaly, tricuspid dysplasia, unguarded tricuspid valve, or leaflet agenesis.

Ebstein’s anomaly is a rare heart disorder and often presents with septal and posterior leaflets adhered to the ventricular wall (i.e., failed delamination), fenestration of the anterior leaflet, downward displacement of the tricuspid annulus, atrialization of the proximal ventricle, and dilation of the atrioventricular orifice and atrialized part of the ventricle [12]. The current donor heart lacked signs of Ebstein’s anomaly. Specifically, the anterior and posterior leaflets appeared typical, the atrioventricular orifice was not displaced, and atrialization of the right ventricle had not occurred.

Tricuspid dysplasia is often mistaken for Ebstein’s anomaly, and an unguarded tricuspid valve has been referred to as a more extreme variant of tricuspid dysplasia [13]. Unlike Ebstein’s anomaly, delamination of leaflets occurs normally in tricuspid dysplasia. However, tricuspid dysplasia is marked by leaflet abnormalities such as prolapse, malformed leaflets (e.g., rolled up, thickened), shortened chordae tendineae, and reduced leaflet mobility [13]. Malformation of leaflets in tricuspid dysplasia results in moderate to severe tricuspid regurgitation [12-13].

Surgical removal of the septal leaflet was also considered because a primary limitation of this case report is the absence of a comprehensive clinical history. The documented cause of death was broadly categorized as “cardiovascular disease,” with no additional diagnostic, operative, or longitudinal medical information available. Although sternal wires were identified on external inspection, the specific prior surgical intervention could not be determined. This raises the possibility of previous cardiac surgery, including procedures involving the tricuspid valve. In the setting of endocarditis or severe tricuspid regurgitation, surgical techniques can effectively “bicuspidalize” the valve, such as the Kay bicuspidalization procedure, where the posterior leaflet is excised [16-17].

Additionally, resection of leaflet tissue may be performed, given the relatively low-pressure nature of the right-sided circulation [16-17]. However, careful gross examination revealed no internal evidence of prior tricuspid valve repair, annular patch material, prosthetic components, or suture lines, nor were there external indicators of prior cannulation or right atrial modification. While the possibility of undocumented surgical alteration cannot be entirely excluded, the absence of identifiable operative changes in addition to the absence of septal papillae and chordae tendineae supports the interpretation of this finding as an isolated congenital absence of the septal leaflet. 

Documented cases of tricuspid valve leaflet agenesis are exceedingly rare in both medical and anatomical literature (Table 1). A focused literature review identified only six previously reported cases: three describing congenital absence of the anterior leaflet and three involving the absence of the posterior leaflet [3-4,6-8,10]. One case of an absent anterior leaflet demonstrated echocardiographic and intraoperative findings comparable to the structural abnormalities observed in this report [8]. To our knowledge, the absence of the septal leaflet in an adult with otherwise intact anterior and posterior leaflets has not been previously described, suggesting a potentially novel anatomic variant.

**Table 1:** Summary of previously reported cases of absent tricuspid leaflets TR: tricuspid regurgitation; TV: tricuspid valve

Leaflet	Age, years	Sex	Presentation	Management	Reference
Anterior	16	M	Severe TV insufficiency	Tricuspid heterograft	[3]
Anterior	46	F	Severe TR	Tricuspid heterograft	[8]
Anterior	10	F	Severe TR, shortness of breath, palpitation, bilateral lower limb swelling	Valve replacement	[10]
Posterior	46	M	Severe TR, atrial septal aneurysm	Posterior annulorrhaphy	[7]
Posterior	28	F	Increasing dyspnea	Tricuspid annuloplasty	[4]
Posterior	62	F	Severe TR	Valve replacement	[6]

Although the cadaveric nature of this case and limited clinical documentation preclude definitive premortem diagnosis, the structural findings provide insight into a potential pathophysiologic progression. The septal leaflet plays a critical role in valve coaptation during systole. Absence of a septal leaflet would predictably prevent complete leaflet apposition, resulting in chronic tricuspid regurgitation similar to previous reports of leaflet agenesis (Table 1). Persistent regurgitant flow from the right ventricle into the right atrium during systole increases right atrial volume and pressure, leading to progressive atrial dilation. Over time, the right ventricle is subjected to sustained volume overload, promoting eccentric hypertrophy and chamber dilation as compensatory mechanisms to maintain forward pulmonary blood flow. Chronic right-sided volume overload may ultimately result in global right heart enlargement, elevated central venous pressures, and secondary structural remodeling [15]. This prolonged hemodynamic burden provides a possible pathway from congenital leaflet absence to right-sided cardiomegaly.

In living patients, tricuspid regurgitation is primarily diagnosed using transthoracic or transesophageal echocardiography [18]. Severity is determined by Doppler-derived parameters, including color flow jet area, vena contracta width, effective regurgitant orifice area, and hepatic vein flow reversal [19]. Management strategies depend on regurgitation severity and associated symptoms, ranging from medical optimization to surgical repair or replacement. Importantly, surgical intervention in the setting of an absent septal leaflet presents unique anatomical considerations. Traditional operative landmarks, such as the Triangle of Koch, which relies in part on the septal leaflet to define its inferior boundary, may be distorted or absent, thereby increasing the risk of inadvertent atrioventricular nodal injury during valve repair or replacement [20].

## Conclusions

Reports describing septal leaflet malformations of the tricuspid valve are exceedingly limited, particularly in cases of complete leaflet absence. This report contributes to existing literature by documenting a previously undescribed variant and outlining a potential pathophysiologic progression from structural anomaly to right-sided volume overload and cardiomegaly. Recognition of such rare anatomical variations is important for cardiothoracic surgeons, interventional cardiologists, and imaging specialists, as altered anatomy may influence diagnostic interpretation, procedural planning, and operative risk. Furthermore, this report underscores the enduring value of body donation in advancing medical knowledge. Careful postmortem investigation continues to provide critical insights into uniquely rare cardiac anomalies such as tricuspid valve agenesis.
